# Dimethyl malonate preserves renal and mitochondrial functions following ischemia-reperfusion via inhibition of succinate dehydrogenase

**DOI:** 10.1016/j.redox.2023.102984

**Published:** 2023-12-05

**Authors:** Mattias Carlström, Lucas Rannier Ribeiro Antonino Carvalho, Drielle Guimaraes, Ariela Boeder, Tomas A Schiffer

**Affiliations:** a Department of Physiology and Pharmacology, Karolinska Institutet, Stockholm, Sweden; b Department of Pharmacology, Federal University of Santa Catarina, Florianópolis, Brazil

**Keywords:** Ischemia-reperfusion, Reverse electron transfer, Dimethyl malonate, Kidney, Glomerular filtration rate

## Abstract

**Background:**

Acute kidney injury (AKI), often experienced at the intensive care units, is associated with high morbidity/mortality where ischemia-reperfusion injury is a main causative factor. Succinate accumulation during ischemia contributes to the excessive generation of reactive oxygen species at reperfusion. Inhibition of succinate dehydrogenase has been associated with protective outcome in cardiac ischemia-reperfusion after 24h, but the effects on kidney and mitochondrial functions are less well studied.

**Aim:**

To investigate the therapeutic potential of succinate dehydrogenase inhibition, by using dimethyl malonate (DMM), on kidney and mitochondria functions in a mouse model of AKI.

**Methods:**

Male C57BL/6J mice were pre-treated with DMM or placebo, *i.p.* 30min prior to bilateral renal ischemia (20min). After 3-days of reperfusion, glomerular filtration rate (GFR) was calculated from plasma clearance of FITC-inulin. Kidney mitochondria was isolated and mass specific and intrinsic mitochondrial function were evaluated by high resolution respirometry. Kidney sections were stained (*i.e.,* hematoxylin-eosin and TUNEL) and analyzed for histopathological evaluation of injuries and apotosis, respectively. NADPH oxidase activity in kidney and human proximal tubular cell-line (HK2) were measured luminometrically.

**Results:**

DMM treatment improved GFR (p < 0.05) and reduced levels of blood urea nitrogen (p < 0.01) compared to untreated animals, which was associated with lower degree of ischemia-reperfusion-induced tubular injuries (P < 0.001) and apoptosis (P < 0.01). These therapeutic renal effects were linked with improved mitochondrial function, both mass-specific and intrinsic. Finally, DMM treatment prevented ischemia-reperfusion-induced NADPH oxidase activity in the kidney (p < 0.001), which was showed also in HK2 cells exposed to hypoxia and reoxygenation (P < 0.01).

**Conclusion:**

Inhibition of succinate dehydrogenase with DMM, in conjunction with the ischemia-reperfusion phase, significantly improved both renal and mitochondrial functions. These findings may have clinical implications for future therapeutic strategies to prevent development of AKI and associated adverse complications, especially in high risk hospitalized patients.

## Highlights

• Acute kidney injury (AKI) is a common adverse complication at intensive care units.
• Ischemia-reperfusion (IR) injury is a major cause of AKI.
• Succinate accumulation and subsequent excessive oxidation lead to a burst of ROS.
• Complex II inhibition in IR preserves kidney and mitochondrial function.
• This finding may important for future preventive strategies of IR-induced AKI.

## Graphical abstract

**Figure:**
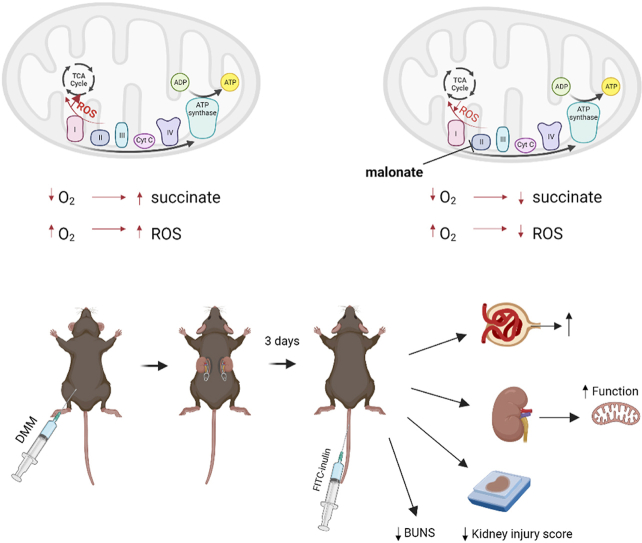

## 1 Introduction

Acute kidney injury (AKI) is a condition that frequently occurs in hospitalized patients. The global burden of AKI-related morbidity and mortality exceeds that of heart failure, diabetes or breast cancer [1]. With ageing populations, the incidence of AKI has significantly increased over the past decades [[2], [3], [4]]. Ischemia-reperfusion injury is the main driving factor causing AKI [5] where conditions such as sepsis, surgery, trauma, nephrotoxic medications, kidney transplantation and heart disease are known inducers of AKI.

Several studies have reported that the risk of experiencing another AKI episode is between 20 and 44 % higher, increasing the risk of progressive chronic kidney disease (CKD) and mortality [[6], [7], [8], [9]]. Identifying new therapeutic strategies that dampen or even prevent ischemia-reperfusion associated AKI are therefore of great importance.

From a pathophysiological perspective, during ischemia, the acidic condition prevents the opening of mitochondrial permeability transition pores (mPTP) [10]. Upon, reperfusion and reoxygenation, a robust generation of oxygen free radicals are produced [11], thus leading to oxidative stress and damage to DNA, lipids and protein [12]. Moreover, this pathophysiological event promotes mPTP opening that aggravates the ischemia-reperfusion injury [13]. Eventually, damage-associated molecular patterns (DAMPs) are released that activate the innate immune response and initiates cytokine release which promote macrophage and neutrophil recruitment [14]. In addition, a crosstalk between mitochondrial and NADPH oxidase (NOX)-derived ROS has been identified leading to a vicious cycle of ROS-induced ROS production at reperfusion [15].

Recently, succinate accumulation in ischemia has been identified as a crucial player contributing to excessive ROS production and detrimental effects at reperfusion [11]. Mechanistically, in ischemia, fumarate overflow from purine nucleotide breakdown and partial reversal of malate/aspartate shuttle in turn contribute to reversal of succinate dehydrogenase (SDH) resulting in succinate accumulation [11]. At reperfusion, succinate is rapidly oxidized and drives the extensive ROS production by reverse electron transfer at mitochondrial complex I (RET) [11]. Thus, succinate dehydrogenase is an obvious target of inhibition to reduce succinate accumulation in ischemia and thus dampen ROS generation at reperfusion.

Indeed, inhibition of SDH using malonate ester prodrugs has previously been shown to be protective in models of cardiac ischemia-reperfusion [[16], [17], [18], [19]], and has also been indicated to offer some protection against renal ischemia-reperfusion injury [20]. Beach and colleagues showed that DMM reduced plasma creatinine levels and mitochondrial ROS after 24h of reperfusion in a mouse model of renal ischemia [20]. However, kidney function (*i.e.*, glomerular filtration rate), mitochondrial respiratory function and histopathological evaluation have not been reported. In addition, the effect of DMM treatment in renal ischemia-reperfusion after a longer time span of reperfusion has not been evaluated.

Here, we aimed to pharmacologically target and inhibit SDH using DMM to evaluate whether this approach may preserve mitochondrial respiratory and kidney functions in a mouse model of AKI, following renal ischemia and three days of reperfusion.

## 2 Methods

### 2.1 Ethics statement

All experimental protocols were approved by the regional Institutional Animal Care and Use Committee in Stockholm (Dnr 17128-2021 and N139/15) and performed according to the US National Institutes of Health guidelines (NIH publication NO. 85-23, revised 1996) and EU directive 2010/63/EU for the conduct of experiments in animals. The experimental approaches for the different *in vitro*, *ex vivo* and *in vivo* experiments are described in detail below.

### 2.2 Animals and kidney ischemia-reperfusion model

Male, 5 months old C57BL/6J mice were obtained from Janvier Laboratories (France) and housed under climate-controlled conditions with a 12-h light/dark cycle and fed with standard rodent chow and tap water ad libitum. Mice were administered saline or dimethyl malonate (DMM) intraperitoneally (300 mg kg^−1^ bw^−1^) 30 min prior to bilateral renal ischemia. Anesthesia was induced and maintained via spontaneous inhalation of isoflurane (3 % for induction and 1.5–2.0 % for maintenance, in medical air) and the body temperature was kept at 37.0 ± 0.5 °C using a self-monitored heating pad together with a heating lamp throughout surgery. Flank incisions were made to allow simultaneous access to both kidneys. The kidneys were inspected and clamped simultaneously for 20 min. Ischemia and reperfusion were confirmed by observing the color change of the kidney. Sham-operated control mice underwent the same surgical protocol but without clamping of the kidneys. Following surgery, the animals were monitored and given buprenorphine two times per day during the first two consecutive days.

### 2.3 Glomerular filtration rate

At the 3rd day of reperfusion, kidney function (determined as GFR) was measured by using plasma clearance of inulin. FITC-inulin (TdB Labs, Uppsala, Sweden) was prepared and diluted in sterile PBS (10 mg/ml) and filtered via a syringe filter (Sartorius AG, minisart, Göttigen, Germany, 0.20 μm). Following tail vein injection of FITC-inulin (100–200 μl), serial blood samples (t = 0, 1, 3, 5, 10, 15, 35, 55, 75 min) were obtained. The syringe was weighted pre and post injection to determine the exact injection volume. Blood samples were taken from the cut tip of the tail into pre-heparinized Eppendorf tubes. After centrifugation, plasma was obtained and protected from light. Samples were diluted in PBS containing 500 mM HEPES (pH 7.4). Fluorescence was measured (excitation/emission 480/530 nm) after loading the sample onto a black 96-well plate using a plate reader (Spectramax iD3, Molecular Devices). FITC-inulin clearance was calculated using non-compartmental pharmacokinetic data analysis as described by Gabrielsson and Weiner [21]. Clearance is defined as the given i.v. FITC-inulin dose divided by the total area under the plasma fluorescence time curve (AUC0-∞).

### 2.4 Histology

Paraformaldehyde-fixed kidneys were dehydrated in increasing concentrations of ethanol, diaffinized by xylol and embedded in liquid paraffin. The kidney tissue blocks were cut by microtome to a thickness of 3 μm. Then, hematoxylin-eosin (HE) stains were performed for evaluation under light microscopy. Tubular injury was scored semi-quantitatively by a blinded histopathologist who examined at least 10 fields from the area of the tubular S3 segment (200× magnification). Tubular injury was defined as tubular dilation, tubular atrophy, tubular cast formation, sloughing of tubular epithelial cells or loss of the brush border and thickening of the tubular basement membrane using the following scoring system: Score 0: no tubular injury; Score 1: <10 % of tubules injured; Score 2: 10–25 % of tubules injured; Score 3: 25–50 % of tubules injured; Score 4: 50–74 % of tubules injured; Score 5: >75 % of tubules injured [22].

### 2.5 Evaluation of apoptosis in kidney tissue

A TUNEL-HRP-DAB assay kit (ab206386; Abcam) was used to assess cell apoptosis in the kidney following ischemia and reperfusion. Kidneys were fixed in 4 % formalin, embedded in paraffin, and cut into 5-μm sections. The staining assay was performed according to the manufacturer's recommendations. Briefly, after deparaffinization and rehydration, the sections were permeabilized with proteinase K for 20 min and endogenous peroxidase activity was suppressed with 3 % H_2_O_2_/methanol for 5 min. The slides were incubated with the TUNEL reaction mixture in a humidified chamber for 90 min. The sections were then blocked with the blocking buffer provided for 10 min and labeled with the streptavidin-horseradish peroxidase (HRP) conjugate diluted 1:25 for 30 min. Diaminobenzidine (DAB) was added to react with the HRP-labeled sample, generating a brown color at the site of the DNA fragments. Methyl green was used as a counterstain. The images were acquired with a light microscope (ZEISS Axioscope, Germany) with a 20× objective. The dark brown staining was quantified using the particle analysis command in the ImageJ software to determine the percentage of positive area.

### 2.6 Blood urea nitrogen

Blood urea nitrogen (BUN), as an alternative approach to assess kidney function and injuries, was measured with a commercial ELISA kit following the manufacturer's instructions (Urea Nitrogen Colorimetric Detection Kit, Thermo Fisher Scientific USA)

### 2.7 Mitochondrial isolation

At the 3rd day of reperfusion, kidneys were extracted, and mitochondria were isolated by differential centrifugation. In brief, tissue was weighted and homogenized using a glass homogenizer (Potter-Elvehjem) in isolation medium containing in mmol L^−1^: 250 sucrose, 10 Hepes, 1 EGTA, BSA 1 g/liter, pH 7,4 compensated with KOH. The homogenate was centrifuged at 700 g for 10 min. Supernatant was collected and recentrifuged at 10000 g for 10 min. Buffy coat on the pellet was carefully removed by pipetting followed by resuspension in isolation buffer. Suspension was centrifuged at 7000 g for 5 min followed by another washing step. Pellet was resuspended in mitochondrial preservation medium (in mmol L^−1^: 5 EGTA, 3 MgCl_2_·6H_2_O, 60 K-lactobionate, 20 taurine, 10 KH_2_PO_4_, 20 HEPES, 110 sucrose, 20 histidine, 3 glutathione, 2 glutamate, 2 malate, 2 Mg-ATP, 1 g L^−1^ BSA, 20 μmol L^−1^ vitamin E succinate, and 1 μmol L^−1^ leupeptin and left to stabilize at least 30 min before respiratory analysis. Pellet was resuspended in 1 μl preservation medium/mg initial sample wet weight.

### 2.8 Mitochondrial respiration

Mitochondrial respiration was evaluated by high resolution respirometry (Oroboros 2K, Innsbruck, Austria). Respiration was normalized to mitochondrial protein or initial wet weight prior to mitochondrial isolation. Preserved mitochondrial integrity was confirmed by measuring respiratory control ratio (RCR) defined as maximal complex I mediated respiratory capacity (pyruvate (5 mM), malate (2 mM) and ADP (2.5 mM)) divided by leak state without adenylates (pyruvate and malate only). Mitochondrial respiration was evaluated in respiration medium containing in mmol L^−1^: 0.5 EGTA, 3 MgCl_2_ * 6H_2_O, 60 K-lactobionate, 20 Taurine, 10 KH_2_PO_4_, 20 HEPES, 110 Sucrose, pH 7.1. LEAK respiration was measured in the presence of pyruvate (5 mM), malate (2 mM) and oligomycin (2.5 μM). Maximal complex I dependent respiration: pyruvate (5 mM), malate (2 mM) ADP (2.5 mM), Maximal CII dependent respiration: succinate (10 mM), ADP (2.5 mM) and rotenone (0.5 μM). Maximal CI + CII dependent respiration: pyruvate, malate, succinate, and ADP. CI proton-leak dependent respiration: pyruvate, malate and oligomycin (10 nM), Maximal CIV activity: ascorbate (2 mM), TMPD (0.5 mM) in presence of antimycin (2.5 μM), ADP (2.5 mM) and cytochrome *c* (10 μM)**.** Correction was made for autoxidation of TMPD/ascorbate at the present oxygen tension. Mitochondrial H_2_O_2_-production was measured using a spectrofluorometric method with amplex red system (5 μM, Amplex ultrared™) together with horseradish peroxidase (1 U/ml) (Sigma-Aldrich P 8250). Calibration of the H_2_O_2_ signal prior to each experiment was performed by adding a standard solution of hydrogen peroxide (180 nmol). Correction was made for autooxidation of Amplex ultrared™.

### 2.9 Kidney tissue citrate synthase activity

A colorimetric citrate synthase (CS) activity kit was obtained from Sigma-Aldrich (MAK193). In brief, kidney tissue was cryo-grinded on liquid nitrogen, followed by dilution in the provided assay buffer and thereafter, the protocol by the manufacturer was followed in detail. CS activity was normalized to protein content and expressed as U/g protein.

### 2.10 Immunoblotting of mitochondrial components of the respiratory chain and NADPH oxidase 4

Kidney tissue samples were cryo-grinded on liquid nitrogen and lysed in RIPA buffer containing protease inhibitor (Sigma-Aldrich, P8340). Samples were briefly sonicated and centrifuged at 14000 g for 15 min. Samples were diluted to a uniform protein concentration and further diluted in commercial Laemmli sample buffer (Bio-Rad, 1:4) containing 10 % 2-mercaptoethanol. Proteins were denatured at 95 °C for 5 min. Samples were separated by electrophoresis on precast gels (Bio-Rad, Criterion™ TGX™, 4–20 %). Transfer to polyvinylidene difluoride membranes was performed using a Bio-Rad, Criterion™ Blotter. Membranes were incubated with primary antibodies overnight at 4 °C followed by secondary antibodies for 1.5 h at room temperature. Primary antibodies used: mouse Anti-OXPHOS antibody cocktail (45–8099, Thermofisher Scientific), rabbit anti-NOX4 antibody (Novus Biological, NB110–58849SS), mouse anti-vinculin (Santa Cruz, sc-25336), HRP-linked secondary antibodies: anti-rabbit (7074S) and anti-mouse (7076S) (Cell signaling Technology, USA), Blots were developed using SuperSignal West Femto Maximum Sensitivity Substrate (Thermo Scientific, Rockford, US). Bands were developed in ChemiDoc MP Imaging System, (Bio-Rad). Proteins were normalized to either total protein (Pierce™ Reversible Stain Kit, Thermo Scientific) or vinculin. Band density was analyzed by using the software Image Lab 6.0.1 (Bio-Rad).

### 2.11 Kidney tissue NADPH oxidase activity

NOX activity was measured in kidney tissue at the 3rd day of reperfusion. Kidney tissue was cryo-grinded on liquid nitrogen and the homogenate was diluted in ice-cold PBS. Samples were centrifuged at 3000 g for 20 min (4 °C). Supernatant was collected for NOX activity measurement. The samples were placed in a luminometer (AutoLumat Plus LB 953, Bethold Technologies) followed by adding lucigenin (5 μM). The samples were subjected to thermal equilibration for 15 min (37 °C). Reaction was initiated by injection of NADPH (100 μM). A kinetic model was applied and the area under curve was calculated in the software GraphPad Prism 9. Results were normalized to protein content.

### 2.12 Cell model to mimic ischemia-reperfusion

Human kidney 2 (HK2) proximal tubular cells were maintained in DMEM/F12 (Gibco) [+] l-glutamine [+] 15 mM HEPES and 10 % FBS, 100 U/ml penicillin, and 100 μg/ml streptomycin. For experimental preparation, cells were cultured at 6-well plates until approximately 75 % confluence, thereafter the cells were washed twice and medium was replaced to Tyrode (in mmol L^−1^:130 NaCl, 5 KCl, 10 HEPES, 1 MgCl_2_, 1.8 CaCl, pH 7.4, 2 DMM in the treated group). Cells were transferred to a hypoxia chamber (Whitley H35 Hypoxy-station) and kept at 0.2 % O_2_, 5 % CO_2_ for 3 h. Before reoxygenation, medium was exchanged to complete medium containing 2 mM DMM in the treated groups. After 1 h of reoxygenation, the DMM containing medium was exchanged to regular complete medium.

### 2.13 NADPH oxidase activity in hypoxia treated HK2 cells

NOX-mediated superoxide production in HK2 cells was evaluated by using lucigenin-derived chemiluminescence, similar to that previously described [23]. Cells were harvested at 16 h post reoxygenation for evaluation of NOX activity. In brief, cell plates were put on ice and washed two times with PBS. After the last washing step, PBS was added to the well and cells were harvested by a cell scraper and resuspended in an eppendorf tube. Cells were transferred to a glass tube followed by addition of lucigenin (50 μM) and put in the luminometer (Berthold Autolomat Plus LB 953) for 15 min thermal equilibration (37 °C). NOX activity was measured by injecting NADPH (Sigma, 100 μM) and the chemiluminescence was measured during 3min. Samples were analyzed using the Software, Berthold version 1.0. The area under curve (AUC) was normalized to cellular protein content and expressed as AUC per mg protein and minute (A.U).

### 2.14 Statistical analysis

GraphPad Prism version 9.2.0 was used for statistical analysis. One-way ANOVA together with Tukey's multiple comparisons test was used to evaluate significant difference between groups. For histopathological statistical difference, non-parametric Mann-Whitney *U* test was used. Data are presented as means ± SD. P < 0.05 was considered significant. * denotes p ≤ 0.05, **p ≤ 0.01, ***p ≤ 0.001 and ****p ≤ 0.0001 respectively. Non-significant differences are indicated as n.s.

## 3 Results

### 3.1 Kidney function

**Fig. 1:**
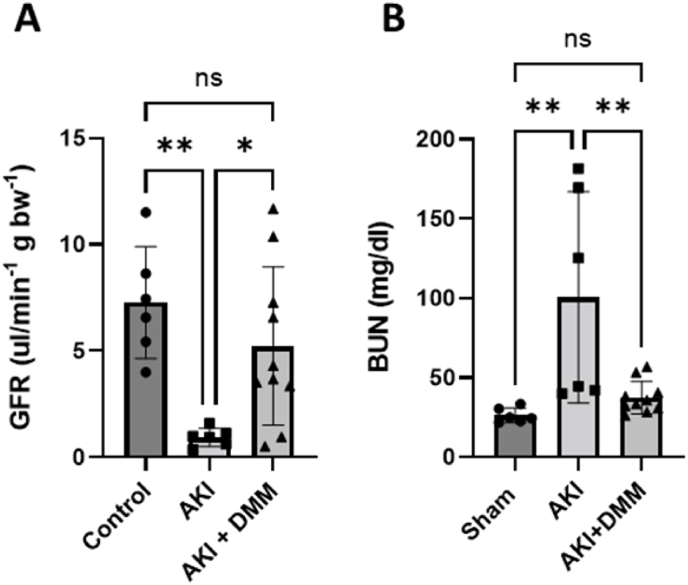
(A), Glomerular filtration rate (GFR) was measured spectrofluorometrically by FITC-inulin injection in the tail vein followed by sequential blood sampling, and (B) blood urea nitrogen (BUN) was measured colorimetrically in mice 3 days after 20 min of bilateral renal ischemia to induce acute kidney injury (AKI) and with/without dimethyl malonate (DMM) treatment. One-way ANOVA was used for statistical analysis.

Glomerular filtration rate (GFR) was used as golden standard to evaluate kidney function. The non-treated group with ischemia-reperfusion showed significant reduction of GFR compared with the sham operated controls (Fig. 1A). Treatment with DMM significantly improved GFR following ischemia-reperfusion and kidney function in this group was not significantly different compared to the sham group (Fig. 1A). In agreement, BUN was significantly increased following ischemia-reperfusion and this was suppressed by DMM treatment to the same levels as observed in the control group (Fig. 1B).

### 3.2 Histopathological evaluation

**Fig. 2:**
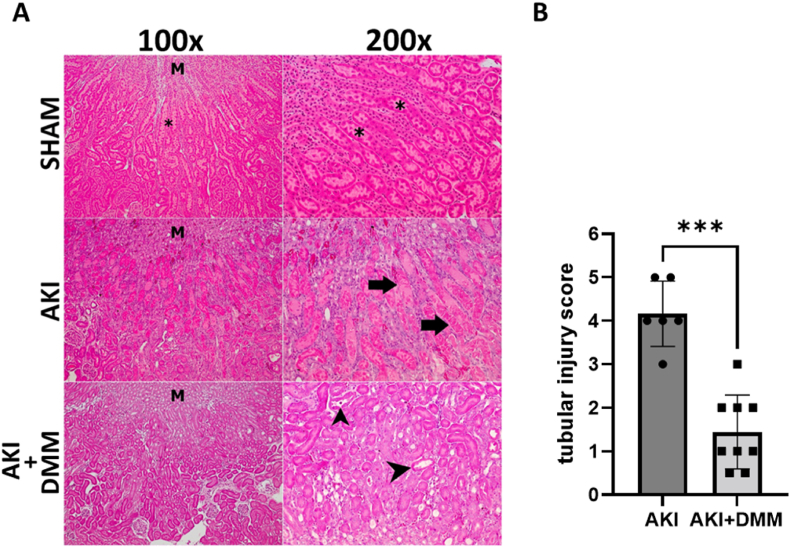
(A) Representative images for histopathological evaluation of paraformaldehyde-fixed mice kidney tissue sections stained with hematoxylin-eosin, analyzed under light microscopy at two magnifications 100× and 200×. Photomicrographs of the S3 segment area with renal medulla as a reference (M), sham group with normal cortical tubules (*), samples from the acute kidney injury (AKI) group with marked cast formation and tubular dilation (arrow) and attenuated injuries in the dimethyl malonate (DMM)-treated group (arrowhead); (B) statistical analysis (Mann-Whitney *U* test) of tubular injury score at the 3rd day of reperfusion in mice treated with DMM prior to ischemia-reperfusion.

As expected, ischemia-reperfusion was associated with marked renal injuries 3 days after the surgery (Fig. 2). The observed tubular dilation, loss of brush border and cast formation in the S3 segment of the proximal tubule of the non-treated ischemia-reperfusion group was significantly attenuated by DMM treatment (Fig. 2A and B). As expected, kidneys of sham-operated controls looked normal and did not show any signs of renal injuries/abnormalities.

### 3.3 Evaluation of apoptotic tissue

**Fig. 3:**
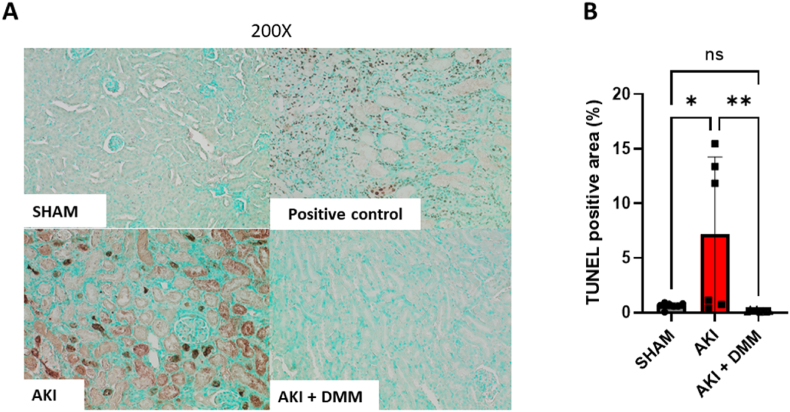
TUNEL-staining was performed in kidney slices following sham or acute kidney injury (AKI). TUNEL-positive area was evalued in by using the software image J. (A) Representative figures of TUNEL stained kidney slices. (B). Statistical analysis of TUNEL positive area at the 3rd day of reperfusion in mice pre-treated with dimethyl malonate (DMM) (One-way ANOVA).

Ischemia-reperfusion is associated with a series of deleterious cellular responses resulting in cell injury and eventual death, where apoptosis is a central part in the pathogenesis of AKI [24]. Results from the TUNEL staining clearly suggested that DMM treatment protected against IR induced apoptosis (Fig. 3A and B).

### 3.4 Mitochondrial function

**Fig. 4:**
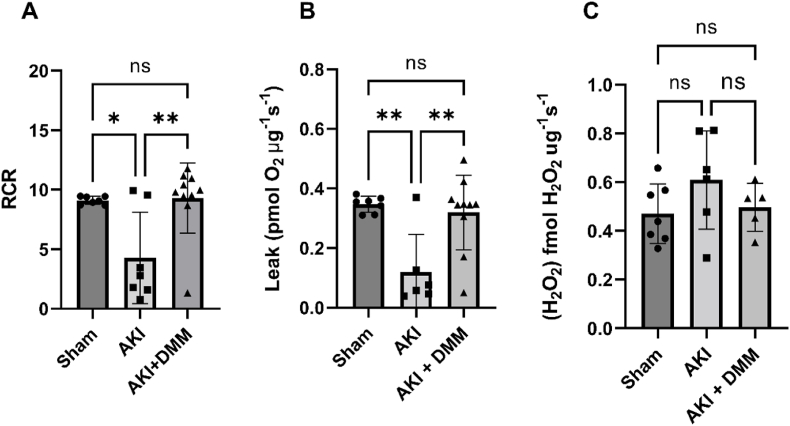
Kidney mitochondrial parameters at the 3rd day of reperfusion (AKI) in isolated mitochondria using high resolution respirometry after treatment with dimethyl malonate (DMM). (A) Mitochondrial respiratory control ratio (RCR), reflecting the degree of coupling/integrity of the mitochondria. (B) Proton leak (across the inner membrane) dependent mitochondrial respiration in presence of complex I substrates and oligomycin. (C) Spectrofluorometric measurement of mitochondrial hydrogen peroxide (H_2_O_2_) production in presence of oligomycin and complex I substrates using the Amplex Ultrared™ system. One-way ANOVA was used for statistical analysis.

**Fig. 5:**
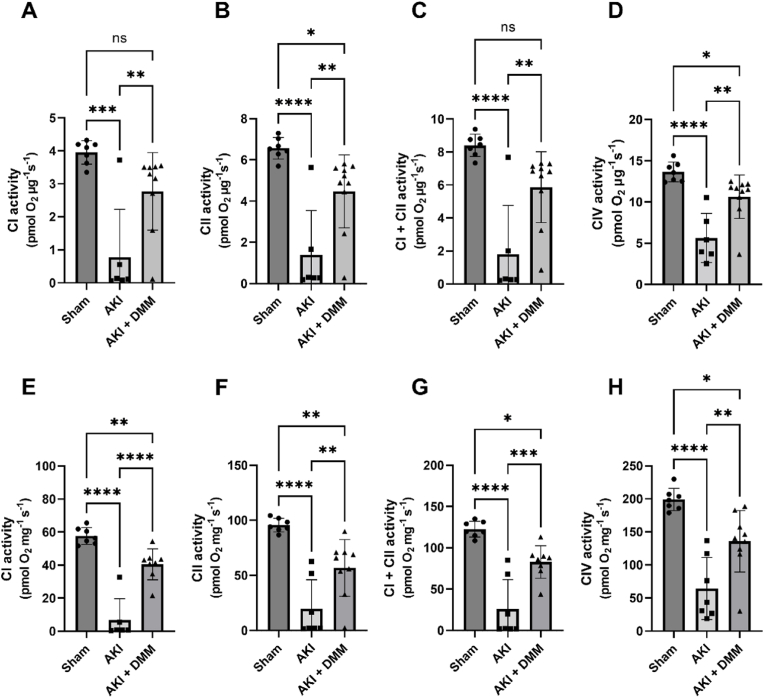
The function of components of the respiratory chain in isolated mitochondria from mice treated with dimethyl malonate (DMM) was measured at the 3rd day of reperfusion (AKI) by high resolution respirometry. Intrinsic mitochondrial function of complex I-IV normalized to mitochondrial protein (A, C, E, G), and mass specific mitochondrial function (CI–CIV) normalized to tissue weight (B, D, F, H). One-way ANOVA was used for statistical analysis.

Considering that mitochondria are vital for maintaining kidney function (*e.g.* providing energy for active transport processes, supporting metabolic homeostasis, participating in oxidative phosphorylation, and influencing cellular survival and apoptosis) we next analyzed mitochondrial parameters using high resolution respirometry. In the kidneys from the non-treated ischemia-reperfusion group, mitochondrial function of all components of the respiratory chain was impaired compared with the sham group (Fig. 5). DMM-dependent succinate dehydrogenase inhibition during ischemia-reperfusion contributed to preserved mitochondrial respiratory control ratio (Fig. 4A). DMM treatment significantly improved both intrinsic (Fig. 5A, C, E, G) and mass specific (Fig. 5B, D, F, H) activity of all respiratory complexes compared to the untreated group. Mitochondrial leak respiration was restored by DMM in the ischemia-reperfusion group (Fig. 4B). Mitochondria-derived hydrogen peroxide production was not significantly changed following ischemia-reperfusion compared with sham-operated mice, and DMM treatment did not contribute to change H_2_O_2_-production (Fig. 4C).

### 3.5 Citrate synthase activity

**Fig. 6:**
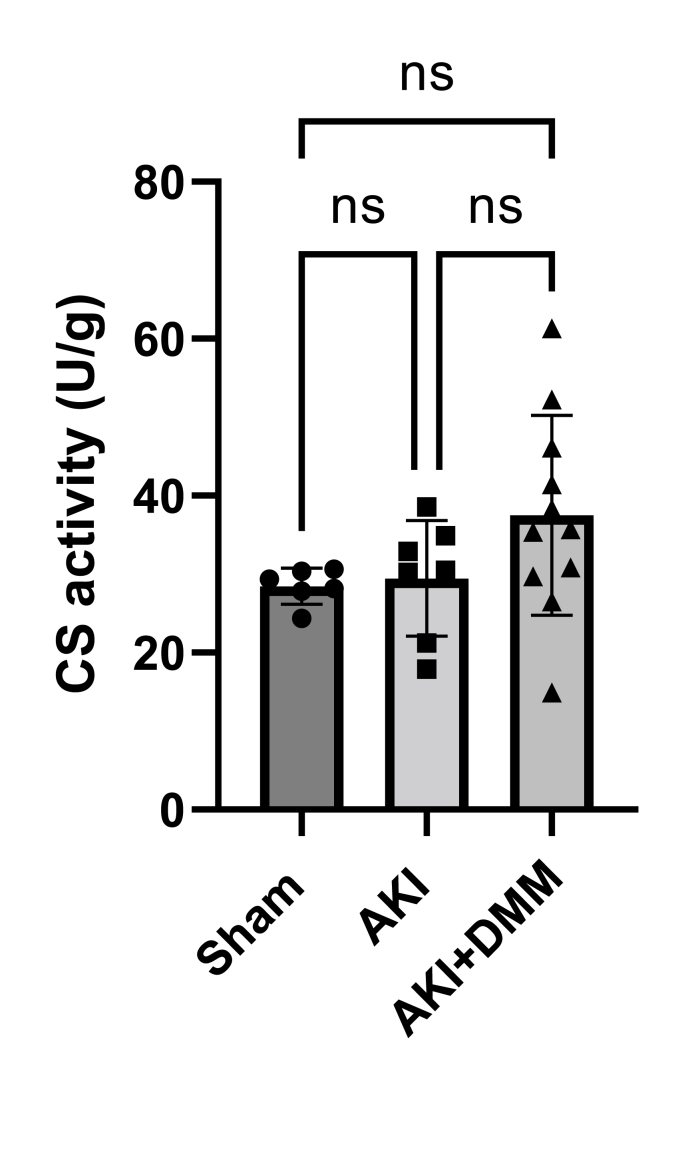
Citrate synthase (CS) activity was measured in cryo-grinded kidney tissue at the 3rd day of reperfusion by using a commercial colorimetric kit. CS activity was normalized to protein content. Acute kidney injury (AKI), dimethyl malonate (DMM). One-way ANOVA was used for statistical analysis.

CS activity is commonly used as a marker of mitochondrial content. However, recent research has questioned that there is a direct relationship between CS activity and mitochondrial content, specifically in kidney [25]. Indeed, here we did not observe any differences in renal CS activity between the treated and untreated groups (Fig. 6).

### 3.6 Oxphos protein levels

**Fig. 7:**
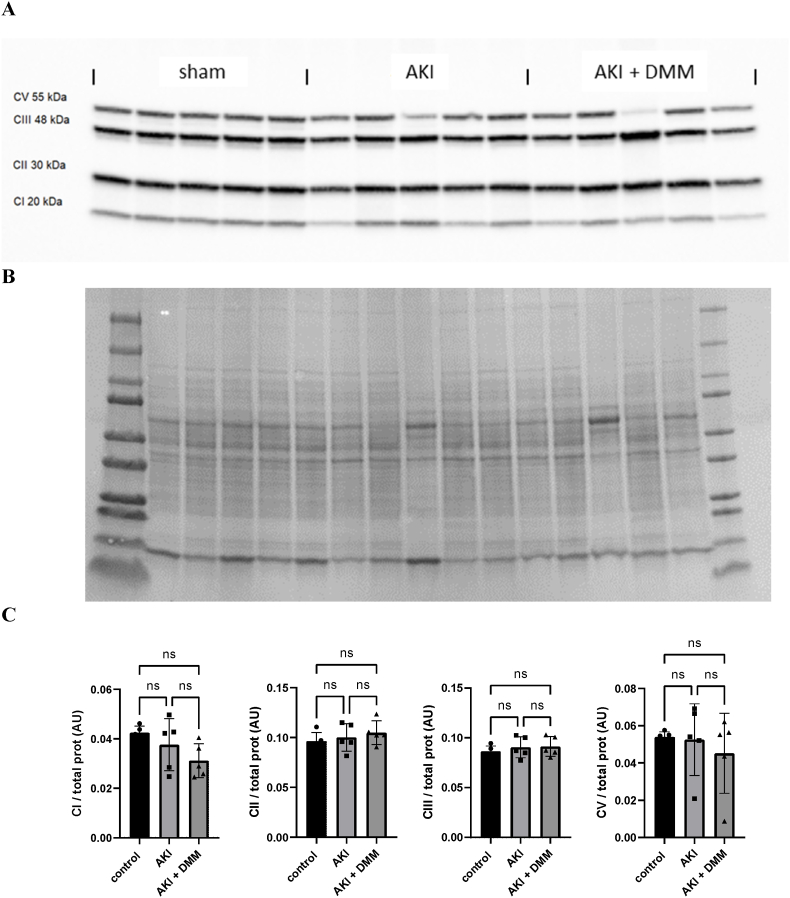
Immunoblotting was performed on components of the respiratory chain with a cocktail of OXPHOS antibodies at the 3rd day of reperfusion (AKI) and treatment with dimethyl malonate (DMM). (A) Protein expression levels of OXPHOS related proteins. (B) Membrane stained for total protein used for normalization. (C) Statistical analysis (One-Way ANOVA) of components of the respiratory chain.

While the ischemic insult highly impaired the mitochondrial respiratory function, protein levels of the components in the respiratory chain did not differ significantly between groups at the 3rd day of reperfusion (Fig. 7A and B).

### 3.7 NADPH oxidase 4 protein levels

**Fig. 8:**
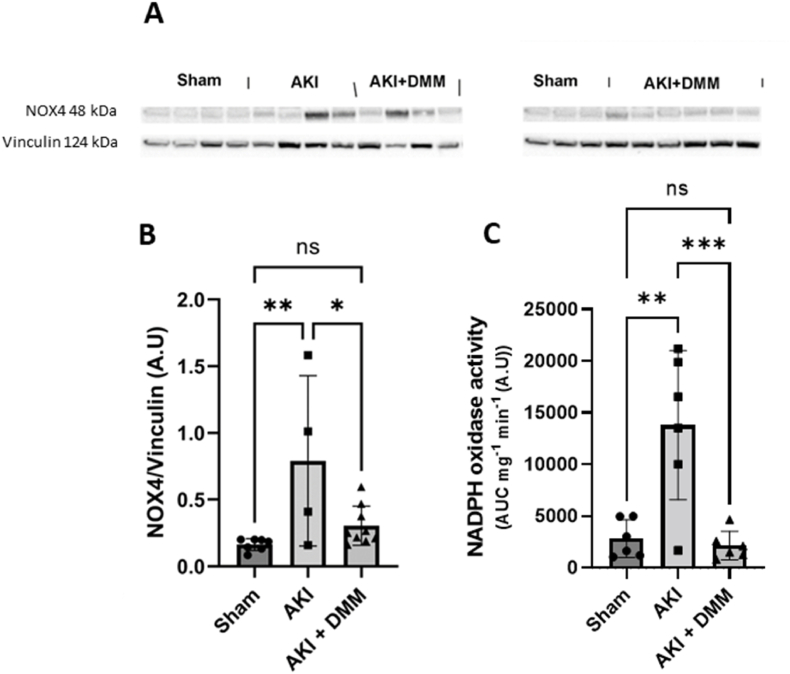
(A) Protein levels of NADPH oxidase 4 (NOX4) were measured by immunoblotting in cryo-grinded kidney samples of mice at the 3rd day of reperfusion (AKI) after pre-treatment with dimethyl malonate (DMM) prior to ischemia reperfusion. (B) Statistical analysis by using one-way ANOVA of the detected NOX4 protein levels. (C) NOX activity was measured in kidney tissue at the 3rd day of reperfusion by using a luminometric method in presence of lucigenin and NADPH. Area under curve was normalized to sample protein content. One-way ANOVA was used for statistical analysis.

While NOX isoforms are primarily associated with the plasma membrane, there is evidence suggesting that NOX4 present in the mitochondria, has been implicated in the pathogenesis of ischemia-reperfusion injury. Here we used Western blot analysis to measure the protein levels of NOX4 in the kidneys for all experimental groups (Fig. 8A). Ischemia-reperfusion *in vivo* was associated with increased NOX4 levels in the kidney, which was significantly supressed by DMM treatment (Fig. 8B). One outlier identified by the software GraphPad Prism 9 was excluded in the AKI + DMM group.

### 3.8 Kidney tissue NADPH oxidase activity

Previous reports observed a major contribution of NOX dependent ROS after an ischemic insult [26]. Indeed, ischemia-reperfusion resulted in significantly increased NOX activity at the 3rd day of reperfusion (Fig. 8C). Interestingly, this was prevented by DMM treatment prior to the ischemic insult (Fig. 8C).

### 3.9 NADPH oxidase acitivity in HK2 cells after hypoxia-reoxygenation

**Fig. 9:**
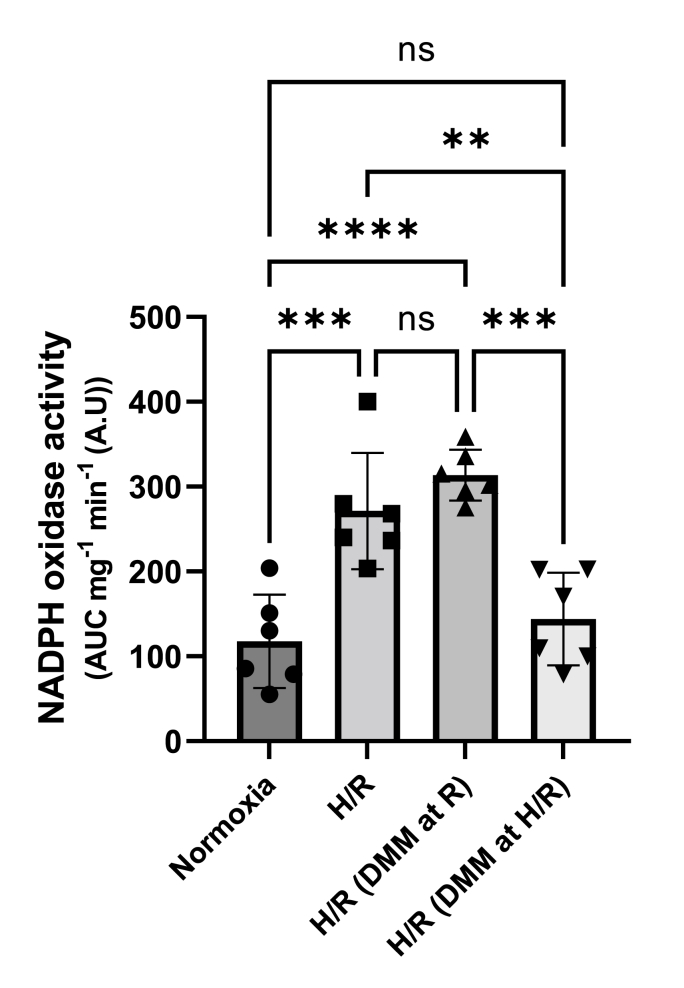
NADPH oxidase (NOX) activity was assessed with lucigenin-derived chemiluminescence in dimethyl malonate (DMM, 2 mM) treated HK2 cells exposed to hypoxia (0.2 % O_2_, 5 % CO_2_) for 3 h and 16 h of reoxygenation (H/R). DMM was present either during hypoxia/reoxygenation (H/R) or at the time of reoxygenation (R). NOX activity is expressed as area under curve (AUC) per mg protein and minute. One-way ANOVA was used for statistical analysis.

As ischemia-reperfusion is associated with increased NOX activity and exaggerated ROS formation, we evaluated the effect of DMM treatment in HK2 cells exposed to hypoxia-reoxygenation, as a model of *in vivo* ischemia-reperfusion. Hypoxia-reoxygenation significantly led to increased NOX activity which was prevented by DMM treatment during ischemia and reoxygenation (Fig. 9). Interestingly, the protective effect of DMM was absent if the drug was added only at reoxygenation (Fig. 9).

## 4 Discussion

Ischemia-reperfusion injury of the kidney is a major clinical problem globally, which contributes to increased morbidity and mortality and is associated with major costs to society. Reduction of glomerular filtration rate (GFR), secondary to kidney injury, is the hallmark of AKI that often progresses to chronic kidney disease (CKD), which in turn contributes to increased risk of cardiovascular co-morbidities [27]. Despite extensive basic and clinical research during the past decades there is still lack of efficient therapeutic strategies that prevent or slow-down the development and progression of ischemia-reperfusion-induced kidney disease [27].

For the first time, we evaluated mitochondrial function and GFR together in a mouse model of AKI and investigated the therapeutic potential of DMM treatment following ischemia-reperfusion. DMM treatment significantly improved GFR, reduced BUN levels (Fig. 1) and improved renal histopathological outcome (Fig. 2).

These favorable effects of DMM treatment on kidney function, observed *in vivo* following ischemia-reperfusion, were accompanied with a preservation of roughly 70 % of both the mass specific and intrinsic mitochondrial function (Fig. 3, Fig. 4).

Surprisingly, citrate synthase activity along with abundance of mitochondrial respiratory components did not differ between groups, indicating that mitochondrial density was not altered after ischemia reperfusion. Recent research indicates a poor correlation between CS activity and mitochondrial content in both kidney and other tissues [25], and therefore a questionable marker of mitochondrial content. In addition, CS activity has frequently been shown to differ in relation to mitochondrial respiratory activity in the pathological situation [28]. Still, the levels of the related proteins of the respiratory chain remained intact, indicative of a reduced respiratory function rather than mitochondrial content. A recent paper show that loss of flavin alters the activity of components of the respiratory chain without affecting the protein content [29]**.**

As the S3 segment of the proximal tubule is most vulnerable to ischemia, the epithelial cell damage appears mainly in this part of the nephron [[30], [31], [32], [33]]. Indeed, we observed tubular dilation, loss of brush border and cast formation in this area following ischemia-reperfusion.

In line with the significantly improved GFR, the histopathological evaluation revealed a profound DMM dependent improvement. By contrast, a previous report using plasma creatinine levels in mice as the sole marker of renal function indicated that inhibition of succinate dehydrogenase offer protection only when administered at reperfusion [20]. Yet, several studies report protective effects in various organs when administered prior to the ischemic event such as rat brain [11], and mouse heart [34].

Moreover, in our cell model of ischemia-reperfusion, the subsequent induction of NOX activity was prevented only in the cells treated with DMM during both hypoxia and reoxygenation (Fig. 9). The discrepancy may be related to different models of ischemia-reperfusion and administration of succinate dehydrogenase inhibitors. Ischemia-reperfusion induced apoptosis has been linked to loss of organ function in AKI [24]. TUNEL staining revealed a profound protection by DMM against AKI (Fig. 3) induced apoptosis which correlates with the observed preservation of mitochondrial function. Mitochondria is a crucial player in apoptotic signaling [35], hence the preserved mitochondrial function may have contributed to the anti-apoptotic effect.

The preservation of mitochondrial function by DMM treatment was likely related to attenuation of succinate accumulation in hypoxia and excessive succinate oxidation-dependent ROS burst at reperfusion [11], inhibiting the formation of mPTP [36] and subsequent vicious cycle of ROS production [37].

Mitochondria together with NOX are the major sources of ROS in relation to AKI [26,38]. Recently, a crosstalk between mitochondrial ROS production and NOX has been established that leads to a feed-forward regulation leading to a vicious cycle of ROS production [15]. Mechanistically, ROS induces opening of mPTP that facilitates the escape of ROS to the cytosol which in turn activates NOX2 in the plasma membrane via PKC and tyrosine kinase [37]. DMM treatment likely prevents or attenuates the formation of mPTP and thereby shuts down the vicious cycle of ROS production. Along with NOX2 upregulation, previous reports also indicate that NOX4 is induced after ischemia-reperfusion via complex interplay of mechanisms that include ROS induced Nf-kB activation [39], hypoxia-induced Hif-1 alpha activation [41] and via complement-dependent activation [40].

Knocking out NOX4 has been shown to offer protection in ischemia-reperfusion of the heart [42]. Our *in vivo* results indicate that succinate dehydrogenase inhibition at ischemia-reperfusion prevents both the induced expression of NOX4 and tissue NOX-activity (Fig. 8), that likely further contributed to the improved outcome.

Surprisingly, recent reports show that even the membrane non-permeable disodium malonate offer protection in ischemia-reperfusion [43,44]. Mechanistically, this was attributed to reduced pH and increased lactate formation which facilitate transport through the sarcolemmal MCT1 in exchange to lactate and further into the mitochondrial matrix via the mitochondrial dicarboxylate carrier [44]. The obvious advantage would be that the drug may specifically target ischemic tissue. Moreover, attention should probably be given towards novel malonate ester drugs such as diacetoxymethyl malonate diester (MAM) that are faster esterase hydrolyzed *in vivo* [16] which may be more efficient in a clinical setting.

## 5 Conclusion

Administration of DMM prior to bilateral renal ischemia preserves both kidney and mitochondrial functions following reperfusion. This suggests that succinate dehydrogenase inhibition in ischemia-reperfusion has potential to prevent the development of AKI in patients at risk, for example undergoing planned surgeries and transplantations.

## 6 Study limitations

The pooled mitochondrial function from homogenates of the whole kidney is a limitation as the mitochondrial function slightly differs between medulla and cortex [45]. However, the majority of kidney mitochondria origin from proximal tubular cells in the cortical and corticomedullary regions [46]. Confirmation of suppression of succinate accumulation in ischemia and attenuation of ROS production at reperfusion *in vivo* was assumed based on previous reports but not measured in this study.

## Declaration of competing interest

The authors declare that they have no known competing financial interests or personal relationships that could have appeared to influence the work reported in this paper.

none
